# A peptide-based approach to evaluate the adaptability of influenza A virus to humans based on its hemagglutinin proteolytic cleavage site

**DOI:** 10.1371/journal.pone.0174827

**Published:** 2017-03-30

**Authors:** Marco R. Straus, Gary R. Whittaker

**Affiliations:** 1 Department of Microbiology and Immunology, College of Veterinary Medicine, Cornell University, Ithaca, New York, United States of America; 2 New York Center of Excellence for Influenza Research and Surveillance, University of Rochester Medical Center, Rochester, New York, United States of America; St. Jude Children's Research Hospital, UNITED STATES

## Abstract

Cleavage activation of the hemagglutinin (HA) protein by host proteases is a crucial step in the infection process of influenza A viruses (IAV). However, IAV exists in eighteen different HA subtypes in nature and their cleavage sites vary considerably. There is uncertainty regarding which specific proteases activate a given HA in the human respiratory tract. Understanding the relationship between different HA subtypes and human-specific proteases will be valuable in assessing the pandemic potential of circulating viruses. Here we utilized fluorogenic peptides mimicking the HA cleavage motif of representative IAV strains causing disease in humans or of zoonotic/pandemic potential and tested them with a range of proteases known to be present in the human respiratory tract. Our results show that peptides from the H1, H2 and H3 subtypes are cleaved efficiently by a wide range of proteases including trypsin, matriptase, human airway tryptase (HAT), kallikrein-related peptidases 5 (KLK5) and 12 (KLK12) and plasmin. Regarding IAVs currently of concern for human adaptation, cleavage site peptides from H10 viruses showed very limited cleavage by respiratory tract proteases. Peptide mimics from H6 viruses showed broader cleavage by respiratory tract proteases, while H5, H7 and H9 subtypes showed variable cleavage; particularly matriptase appeared to be a key protease capable of activating IAVs. We also tested HA substrate specificity of Factor Xa, a protease required for HA cleavage in chicken embryos and relevant for influenza virus production in eggs. Overall our data provide novel tool allowing the assessment of human adaptation of IAV HA subtypes.

## Introduction

Influenza A viruses (IAV) belong to the class of *Orthomyxoviridae* and it is believed that waterfowl are their natural reservoir. There are 18 hemagglutinin (HA) and 9 neuraminidase (NA) subtypes in nature, with H1–H16 circulating in wild birds [1,2]. In wild birds, the virus typically replicates in the intestine without causing noticeable disease symptoms in its natural host [3]. Infected birds, however, are able to spread disease to poultry where the virus typically has respiratory tropism but can mutate to a more highly pathogenic form, resulting in the culling of millions of birds and significant economic losses [4]. Moreover, such outbreaks expose humans to avian IAV on a large scale, and drastically increase the risk of human infections. In humans, this can result in devastating outbreaks with high mortality rates such as the “Spanish flu” of 1918, the “Asian pandemic” of 1957 or the “Hong Kong pandemic” of 1968 caused by H1N1, H2N2 and H3N2, respectively [3]. Moreover, in 2009 H1N1 was responsible for the first pandemic of the 21^st^ century [5]. Besides H1, H2 and H3, only a few other subtypes have emerged that were shown to infect humans. To date, H5N1, H6N1, H7 in combination with various NA subtypes, H9N2 and H10N8 strains have caused disease and fatalities in human populations [6–14].

Early studies showed that IAVs can undergo replication in the absence of exogenous proteases [15,16]. However, addition of trypsin/plasmin results in a significant enhancement of IAV infectivity. Therefore, activation of HA seems to be a crucial virulence factor in the life cycle of IAV and in host adaptation. In order to enter host cells, the viral HA protein binds to sialic acid receptors on the host cell membrane, which leads to the endocytosis of the virus [17,18]. Once in the endosome, the acidic environment causes a conformational change of the HA protein thereby exposing the fusion peptide, followed by the fusion of viral and cellular membranes and the release of the viral RNA into the host cell. Certain adaptations such as lowering the fusion pH and change in receptor binding preference from α(2,3)-linked to α(2,6)-linked sialic acid allow the virus to cross species barriers and to infect mammalian hosts [19,20]. In order to become pandemic, IAVs also require the acquisition of airborne transmission which seems to be facilitated by a set of complex genetic changes that are not yet fully understood [20–22].

HA is synthesized as a precursor that needs to be cleaved by host proteases to exert its fusogenic activity [23,24]. Cleavage occurs at the C-terminal side of a single arginine residue, which is preceded by an amino acid sequence that is very variable among different viruses and impacts the virulence of a given strain [17,23]. Low pathogenicity avian influenza (LPAI) viruses possess a “monobasic” cleavage site that consists of 1–2 non-consecutive basic amino acids and are likely processed by trypsin in the gastro-intestinal tract in their natural host. In humans or poultry, equivalent viruses are processed by trypsin-like serine proteases in the respiratory tract, which are either membrane bound or secreted into the extracellular space [17,23,25,26]. As these proteases are restricted to the respiratory tract, infectivity is mainly confined to this tissue. Several type II transmembrane serine proteases (TTSPs) in the human respiratory tract have been identified to cleave HA proteins, including TMPRSS2, TMPRSS4, human-airway tryptase (HAT) and matriptase, as well as a number of secreted proteases such the kallikrein-related peptidases 5 (KLK5) and 12 (KLK12) and plasmin [27–32]. Of the membrane-bound proteases, some (e.g. matriptase) can also cleave in a secreted form lacking a transmembrane anchor, but others (e.g. TMPRSS2) only functionally cleave HA in a membrane bound form [33,34]. In contrast to LPAI, highly pathogenic avian influenza (HPAI) viruses are characterized by a polybasic cleavage site (typically 6–7 basic residues) allowing them to be cleaved by ubiquitous furin-like serine proteases such as furin or PC6 [35]. These enzymes are not restricted to a particular tissue and drastically increase the risk of a systemic IAV infection. However, which proteases cleave HPAI in the human respiratory tract is not clear. A noteworthy non-human protease involved in the cleavage of HA is Factor Xa [36,37]. Factor Xa has been shown to activate H1N1 HA in embryonated chicken eggs which is still the major source for IAV production to generate vaccines.

While various studies have described the proteolytic activity of individual human proteases towards a specific HA subtype [27–31,38,39], there is only one study by Galloway et al. that examined the cleavage pattern of representative HA proteins of all subtypes [27]. The study was, however, limited to only two proteases present in the human respiratory tract, TMPRSS2 and HAT. Given the public health threat that IAV poses, we investigated the HA subtype specificity of relevant human proteases towards IAV strains that have caused pandemics in the past, or are currently of major concern for human adaptation. We generated peptides that mimic the cleavage site of H1, H2, H3, H5 (LPAI and HPAI), H6, H7, H9 and H10 strains and which are flanked by a FRET pair (see Experimental Procedures section for details). Cleavage of the peptide results in the separation of acceptor and quencher molecule and hence allows a quantitative measurement of the proteolytic activity of the tested enzymes. In this assay we used trypsin, HAT, the kallikrein-related petidases 5 and 12, the secreted catalytic domain of matriptase, plasmin, furin and factor Xa. Compared to *in vivo* experiments using full length HA proteins *in vitro* peptide assays provide a rapid and quantitative method to investigate HA subtype cleavability as a tool to assess the potential of emerging or pandemic IAV strains to adapt to humans.

## Materials and methods

### Proteases and peptides

Trypsin and plasmin were obtained from Sigma-Aldrich. The catalytic domain from matriptase, recombinant HAT, KLK5 and KLK12 were purchased from R&D Systems. Furin and Factor Xa were obtained from NEB. All peptides listed in Table 1 were synthesized by Biomatik to a purity of at least 95%. The peptides were modified with the fluorescence resonance energy transfer (FRET) pair 7-methoxycoumarin-4-yl acetyl (MCA) at the N-terminus and N-2,4-dinitrophenyl (DNP) at the C-terminus. The final concentration of each peptide was 50μM per reaction.

**Table 1:** List of the different HA subtypes and subtype isolates with the corresponding peptide sequence used for the cleavage assays. P1, P2, P3 and P4 designate the amino acid residues next to the the cleaved bond towards the N-terminus with P1 being the residue proximal to the cleavage site [40].

HA subtype	Representative Strain	N-terminus	P4	P3	P2	P1	C-terminus
H1	A/California/04/2009 H1N1	(MCA)^a^-IPS	I	Q	S	R	GL-(DNP)^a^
H1	A/WSN/1933 H1N1	(MCA)-IPS	I	Q	Y	R	GL-(DNP)
H1 S343G	/	(MCA)-IPS	I	Q	G	R	GL-(DNP)
H2	A/Japan/305/1957 H2N2	(MCA)-VPG	I	E	S	R	GL-(DNP)
H3	A/Hong Kong/1/1968 H3N2	(MCA)-VPE	K	Q	T	R	GL-(DNP)
H5	A/Vietnam/1203/2004 H5N1 LPAI	(MCA)-VPQ	R	E	T	R	GL-(DNP)
H5	A/Vietnam/1204/2004 H5N1 HPAI	(MCA)-EKR	R	K	K	R	GL-(DNP)
H6	A/Taiwan/2/2013 H6N1	(MCA)-VPQ	I	A	T	R	GL-(DNP)
H6	A/quail/Hong Kong/17-21-30/1999 H6N1	(MCA)-VPQ	I	E	T	R	GL-(DNP)
H7	A/Shanghai/02/2013 H7N9	(MCA)-PEI	P	K	G	R	GL-(DNP)
H7	A/Chicken/New York/13833/1995 H7N2	(MCA)-PEI	P	K	P	R	GL-(DNP)
H7	A/turkey/UT/24721/-10/1995 H7N3	(MCA)-PEI	P	K	T	R	GL-(DNP)
H9	A/Hong Kong/2108/2003 H9N2	(MCA)-VPA	R	S	S	R	GL-(DNP)
H10	A/Duck/Shanghai/602/2009 H10N8	(MCA)-PEL	M	Q	G	R	GL-(DNP)
Optimized Furin Substrate	/	(MCA)-AAS	R	S	R	R	SAA-(DNP)

^a^ MCA = 7-methoxycoumarin-4-yl acetyl and DNP = N-2,4-dinitrophenyl.

### Buffers

Trypsin was diluted in 20 mM Tris pH 7.5 and 150 mM NaCl. HAT was diluted in 50 mM Tris pH 9.5 and 0.05% Brij-35. Matriptase was diluted in 50 mM Tris pH 9, 50 mM NaCl and 0.01% Tween. KLK5 and KLK12 were diluted in 100 mM Tris pH 7.5, 150 mM NaCl, 10 mM CaCl_2_ and 0.05% Brij-35. Plasmin was diluted in 50 mM Tris pH 7.4 and 50 mM NaCl. Furin was diluted in 100 mM HEPES, 1 mM CaCl_2_, 1 mM 2-Mercaptoethanol and 5% Triton X-100. Factor Xa was diluted in 20 mM Tris pH 8, 100 mM NaCl and 2 mM CaCl_2_. KLK12 was incubated in activation buffer (100 mM Tris pH 8, 150 mM NaCl, 10 mM CaCl_2_, 0.05% Brij-35) for 24 h prior to the cleavage assay.

### Peptide cleavage assay

All enzymes and peptides were diluted in the respective enzyme buffer to a final concentration of: 0.8 nM trypsin, 5 nM HAT, 40 nM matriptase, 20 nM KLK5, 20 nM KLK12, 0.0025 U plasmin, 1 U furin, 0.5 μg Factor Xa, 50 μM peptides. The reactions were carried out at 37°C and cleavage was measured as a change of fluorescence at 390nm (SpectraMax GeminiXPS, Molecular Devices). V_max_ values were calculated using Microsoft Excel and data was plotted using GraphPad Prism 7 software.

## Results

### HA cleavage profile of the pandemic H1, H2 and H3 strains

To establish our assay, we first examined the cleavage profile of influenza viruses known to circulate in the human population (H1, H2 and H3), using a range of soluble proteases previously reported to activate the viral HA protein.

#### H1 HA

**Fig 1:**
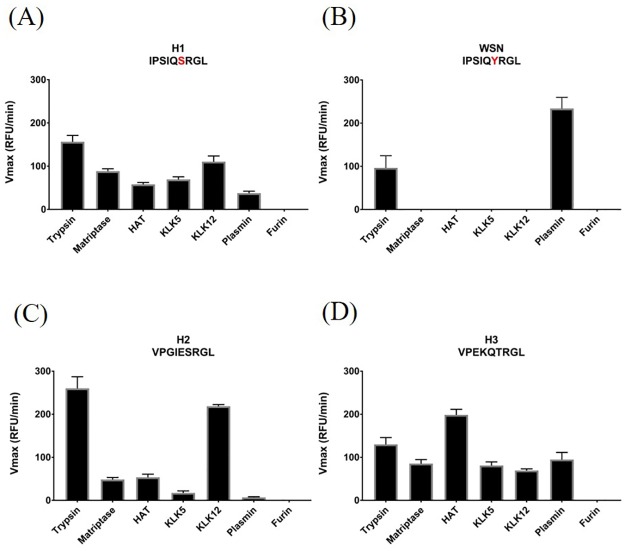
Comparison of HA subtypes that caused pandemics. (A) Cleavage site mimetic peptide representative of H1N1 strains was monitored for cleavage by the indicated proteases. Red letter in the peptide sequence emphasizes a different amino acid compared to the cleavage sequence of the H1 isolate A/WSN/33 (B). (C) and (D) display cleavage assays of peptide mimics representative for H2N2/H2N6 and H3N2 strains, respectively. RFU—relative fluorescence units.

In 1918, an H1N1 influenza virus was responsible for one of the most devastating pandemics in modern human history and since then H1N1 viruses have caused two more outbreaks in 1977 and 2009 [41,42]. There is evidence that the three H1 viruses have evolved from different sources, however, they all share the same HA cleavage site sequence (Table 1) [20,43,44]. While the 1918 and 1977 IAV strains have disappeared, the 2009 H1N1 virus is still circulating in human populations and poses a threat to public health. We tested the cleavability of H1N1 HA and found that, with the exception of furin, all proteases in our assay cleaved it very efficiently (Fig 1A). Hence, H1N1 viruses appear to be cleaved by a broad range of host proteases. In contrast, the HA of the highly laboratory- and mouse-adapted, and neurovirulent, IAV strain A/WSN/33 (which carries Tyr instead of Ser in the P2 position) is cleaved only by trypsin and plasmin and not by the respiratory tract proteases tested (Fig 1B).

#### H2 HA

Influenza H2N2 viruses caused a global pandemic in 1957 that resulted in approx. two million deaths, but since then H2N2 influenza has disappeared from circulation [3,42]. In 2006, a novel H2N3 virus was identified that can be transmitted between pigs and ferrets and which shares the same HA cleavage site as the 1957 pandemic strain [45]. Even though H2N3 is currently only circulating in birds, transmissibility among mammals raises the concern of its pandemic potential. When we tested the ability of different proteases to cleave H2 HA, we found a similar result as with H1 HA (Fig 1A and 1C). All proteases except furin were able to proteolytically process H2 HA. Compared to H1, however, KLK12 had a much higher affinity towards the H2 HA substrate while matriptase, HAT, KLK5 and plasmin cleaved it less efficiently.

#### H3 HA

The third global pandemic of the 20^th^ century was caused by H3N2, which killed about one million people worldwide [42]. The virus has been circulating in humans since then with recent high incidence levels in 2011 and 2012 [42,46,47]. In our peptide cleavage assay, H3 showed a similar profile as H1 (Fig 1D). All proteases except furin cleaved H3 HA efficiently, with HAT expressing the highest activity towards this substrate.

### Cleavage assay of a novel H10N8 HA subtype

**Fig 2:**
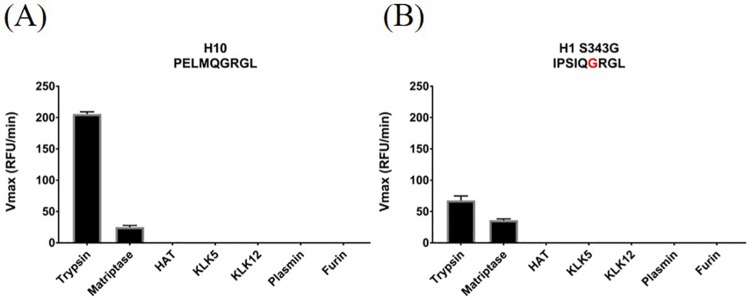
Cleavage profile of the H10 subtype. (A) The peptide mimicking the cleavage motif of the H10N8 strain was incubated with the indicated proteases and cleavage was monitored by the increase of fluorescence at 390 nm. Red indicates the amino acid change that was created in the H1 peptide mimic to resemble the H10 sequence (B).

H10N8 influenza was first detected in humans in 2013 [14]. To date, three cases have been reported of which two had a lethal outcome. However, evidence points to an earlier adaption of H10N8 to mammals when the virus was detected in feral dogs living in proximity to poultry markets in China [48]. Recent studies provided evidence that H10N8 IAV has not yet evolved a preference for human receptor binding [49,50]. Currently, H10N8 is under high surveillance because of its potential to infect humans [51,52]. Our data reveal that the H10 HA subtype is cleaved only very poorly by the human respiratory tract proteases tested (Fig 2A). Trypsin was able to cleave H10 HA efficiently and matriptase expressed a low activity towards the peptide substrate, but none of the other proteases showed any detectable activity. In order to understand why the cleavability of H10 HA is significantly compromised, we created a mutated version of the H1 peptide that carried Gly instead of Ser in the P2 position and therefore resembled the H10 peptide (Table 1). The cleavage pattern of the mutated H1 peptide was very similar to the one observed with H10, and ubiquitous cleavage seen with the native H1 sequence was abrogated (Fig 2B). This therefore suggests that the sequence Gln-Gly-Arg in the P3, P2 and P1 positions, respectively, is not optimal for proteolytic processing by most human respiratory tract proteases.

### Distinct cleavage patterns in H6N1 variants

**Fig 3:**
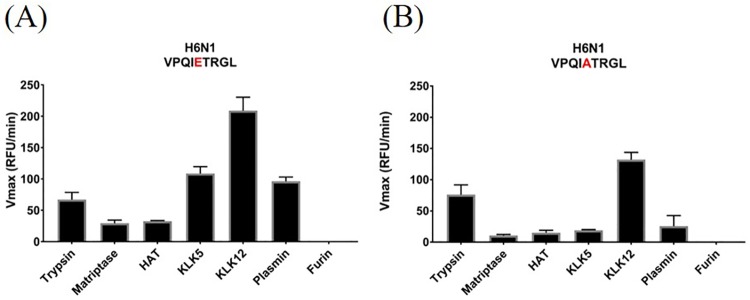
Differences in cleavage pattern of two H6N1 isolates. (A) and (B) show the cleavage site sequence of two H6N1 strains circulating in the early 21^st^ century and more recent ones, respectively. Red letter indicates the changed amino acid at the P3 position in the cleavage site.

Another virus under high surveillance interest because of its potential to infect humans is H6N1. In 2013, the first case of a human H6N1 infection was reported in Taiwan [8]. Recently, studies have shown that the identified A/Taiwan/2/2013 strain exhibits increased binding to mammalian receptors and that H6N1 strains have adapted to human receptors over the past 20 years [53,54]. Moreover, recent H6N1 strains seem to have established a change in the cleavage motif, carrying Ala in the P3 position instead of Glu [8]. We were interested to see whether this amino acid change results in a distinct cleavage pattern that may reflect the increased pathogenicity of H6N1. Both H6 peptides were cleaved by all tested proteases (Fig 3A and 3B). While KLK5, KLK12 and plasmin cleaved the IETR peptide very efficiently (Fig 3A) their activity towards the IATR peptide was strongly abrogated (KLK5 and plasmin) or attenuated (KLK12). However, matriptase and HAT processed both substrates with a low efficiency (Fig 3A and 3B). Overall, these data suggest that recent H6N1 viruses are less efficiently cleaved by human-specific proteases than earlier isolates.

### Differences in the cleavage of H5N1 LPAI and HPAI HA subtypes

**Fig 4:**
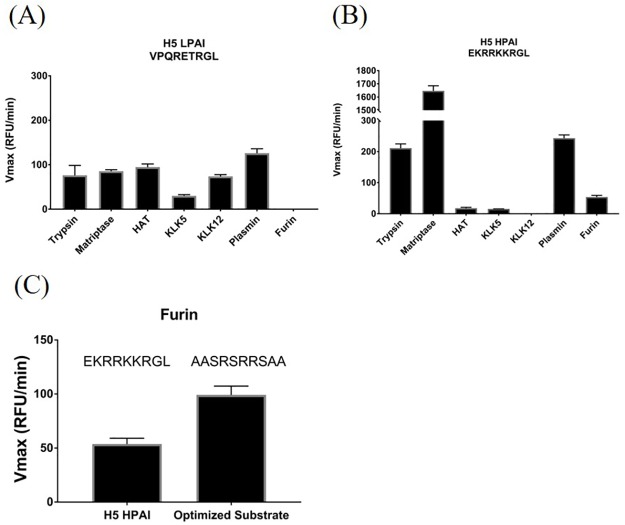
Comparison of H5N1 LPAI and HPAI subtypes. Peptides mimicking the LPAI sequence (A) and the HPAI sequence (B) of H5N1 were treated with the indicated proteases. (C) Furin was incubated with the H5N1 HPAI peptide mimic on the left and a designed control peptide which carries an optimal furin cleavage site on the right.

H5N1 strains circulate in waterfowl as LPAI, and evidence suggests that they evolve to HPAI in infected poultry [55]. There are reports of infrequent direct avian to human transmission of H5N1 HPAI, resulting in mortality rates of about 60%. However the potential of human to human transmission seems very limited and not sustainable [56,57]. Recent outbreaks in Egypt, however, have raised public health concerns because circulating strains have acquired mutations that promote transmissibility in ferrets and allow efficient replication in mammals [58]. We examined both H5N1 LPAI and HPAI for the cleavability by human-specific proteases. The H5N1 LPAI HA subtype was cleaved by all tested proteases, except furin (Fig 4A). The H5N1 HPAI HA subtype, however, was cleaved more efficiently by trypsin, matriptase, plasmin and furin (Fig 4B). Cleavage by HAT, KLK5 and KLK12 was strongly attenuated for HPAI. It is noteworthy that matriptase expressed an approx. 16-fold higher activity with the polybasic HPAI cleavage site compared to the LPAI motif. This activity (Vmax 1650 RFU/min) was also approximately 30-fold higher than with furin, the protease expected to cleave HPAI. Furin, which is known to require paired basic residues in its substrate, had a Vmax of only 53.79 RFU/min (S1 Table) with HPAI. The polybasic cleavage motif in HPAI H5N1 does not represent an optimal furin cleavage site. Furin preferentially cleaves an Arg-X-Arg/Lys-Arg motif and prefers flanking serine residues [59], whereas HPAI H5N1 carries an Arg-Arg-Arg-Lys-Lys-Arg | Gly cleavage site [60]. In order to understand how efficiently the H5N1 HPAI peptide is cleaved by furin we compared it to a designed peptide that has an optimal furin cleavage site (Table 1, Fig 4C). The Vmax for the optimized substrate was 99.28 RFU/min and therefore about two-fold higher as observed for the H5N1 HPAI peptide (Fig 4C, S1 Table). These data suggest that HPAI, while having a functional furin cleavage site, may be preferentially cleaved by certain respiratory tract proteases, such as matriptase.

### Cleavage profiles of distinct IAV H7 subtypes

**Fig 5:**
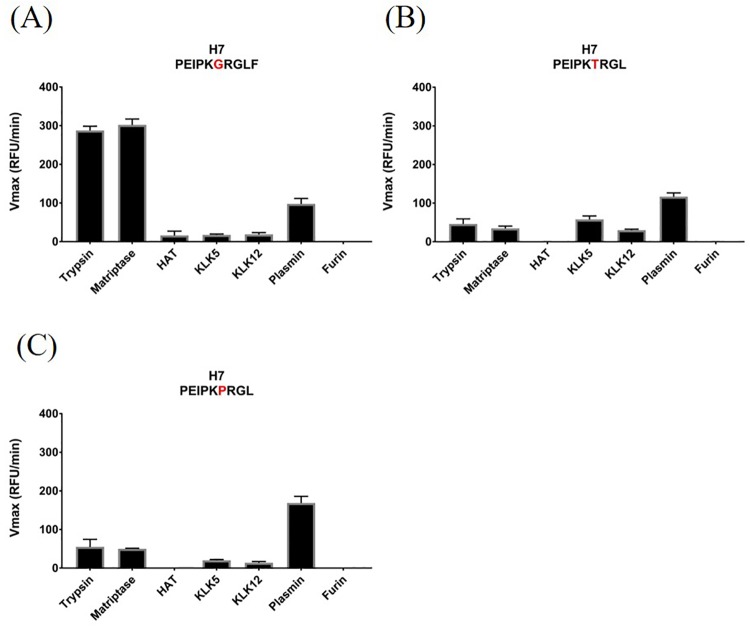
Cleavage profile of H7 isolates. Cleavage rates of three different peptides representing the HA subtypes of the H7N9 (A), H7N3 (B) and H7N2 (C) isolates were analyzed. Cleavage by the indicated proteases was monitored by the increase of fluorescence at 390 nm. Red letters in the peptide sequence highlight the distinct amino acid at the P2 position in the different isolates.

Infections in humans with H7 influenza A subtypes have been reported since the 1970s [3]. This included infections with H7N2 and H7N3 which mostly resulted in conjunctivitis and only one documented fatality [3,61]. Recently, a novel H7N9 strain evolved which infected several hundreds of people in China with a case fatality rate of 30% and which is a major concern for public health [9,62]. The various H7 HA subtypes differ at the P2 position in their cleavage motif (Table 1). Therefore, we tested the three H7 LPAI motifs which have been identified to date in our protease panel to investigate whether the cleavage profile provides insight into the virulence of a given strain. The H7N9 HA subtype carrying Gly in the P2 position was cleaved by all tested proteases with the exception of furin (Fig 5A). Instead of Gly, H7N3 and H7N2 have Thr and Pro at the P2 position, respectively (Table 1). The cleavage profiles for the H7N3 (Fig 5B) and H7N2 motifs (Fig 5C), however, resemble the pattern observed with the H7N9 subtype with two major differences. First, the affinity of trypsin and matriptase for H7N9 is about 7-10-fold higher than for the other two substrates. And second, HAT did not cleave the H7N2 and H7N3 subtypes. As with H5N1, these data suggest that matriptase may be an important protease for activation of H7N9 influenza in humans.

### H9N2 cleavage assay

**Fig 6:**
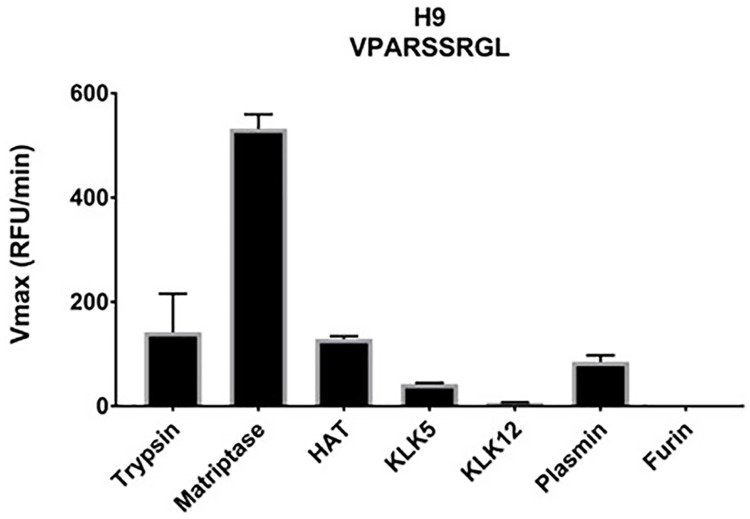
Analysis of H9N2 subtype. A peptide mimetic representative of the H9N2 strain was incubated with the indicated proteases and cleavage was measured as an increase of fluorescence at 390 nm.

The first human infection with H9N2 was reported in 1997 and was caused by a virus lineage that was genetically related to isolates from human H5N1 IAV [63]. There have been very few other documented cases of human H9N2 infections and to date there is no evidence for the transmissibility among humans. However, its relation to human H5N1 and the emergence of H9N2 isolates with dual or human-like sialic acid receptor specificity classify H9N2 as a strain with a high potential to infect humans [64]. We found that the H9N2 HA subtype was cleaved well by trypsin, matriptase, HAT and plasmin (Fig 6). KLK5 showed little affinity towards the substrate while KLK12 barely cleaved it and furin did not express any activity. As previously observed with H5N1 HPAI and H7N9 HA subtypes matriptase cleaves H9N2 very efficiently (Figs 4B, 5A and 6).

### Cleavage specificity of Factor Xa towards different HA subtypes

**Fig 7:**
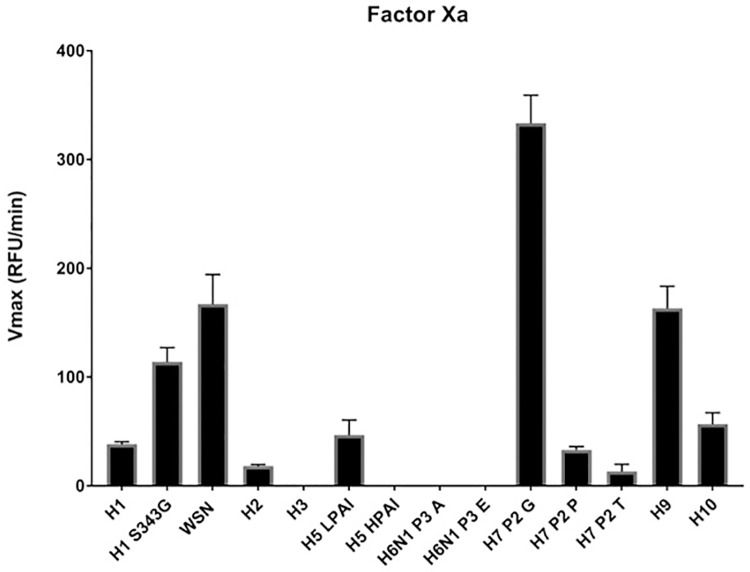
Cleavage analysis of distinct HA subtypes by the Factor Xa protease. The different peptides mimicking the cleavage motifs of the indicated HA subtypes were incubated with Factor Xa and the cleavage was monitored by the increase of fluorescence at 390 nm. H6N1 P3 E and H6N1 P3 A designate the H6N1 peptides that carry Glu and Ala at the P3 position, respectively. H7 P2 G, H7 P2 P and H7 P2 T indicate peptides that have Gly, Pro or Thr in the P2 position, respectively.

Propagation of IAVs in embryonated chicken eggs is currently still the prevailing method for vaccine production. Factor Xa has been identified as the major protease that cleaves HA substrates preferentially at a Ile-Glu/Asp-Gly-Arg motif [36,37]. Published data, however, is confined to experiments performed with H1 HA subtypes and it was reported that certain IAVs do not propagate well in embryonated chicken eggs [65,66]. We therefore tested the specificity of Factor Xa towards different HA subtypes. Interestingly, most of the IAV strains with a potential to infect humans such as H1N1, H2N2, H3N2, H5N1 and H10N8 were cleaved very poorly or not at all (Fig 7). Some of the lower risk strains such as H6N1, H7N2 and H7N3 were also not cleaved or only very inefficiently. Proteolytic processing improved for H1N1 when we exchanged the P3 residue from Ser to Gly, which then was similar to the preferred Factor Xa cleavage motif. However, the best cleavage was achieved with WSN, H9N2 and H7N9, substrates that do not resemble a prototypical Factor Xa cleavage site.

## Discussion

Cleavage activation of HA by host proteases is a critical and crucial step in the life cycle of IAV that ultimately impacts on its virulence, transmissibility between hosts and adaptation to new host species. Several host proteases in humans have been identified to cleave HA *in vitro* and/or *in vivo*. However, data describing the specificity of these proteases towards different HA subtypes is very limited. The most comprehensive study by Galloway et al., analyzed the cleavage of 16 representative HA subtypes but was restricted to two proteases present in the human respiratory tract [27]. Here, we describe the cleavage specificity and pattern of eight proteases by screening 14 different peptides that mimic the cleavage motif of distinct HA subtypes. Our analysis provides a rapid and quantitative tool to assess the cleavability of HA. Moreover, our screen is able to contribute significant data that will help to assess the potential of human adaptability of currently circulating IAV strains.

The structure of the cleavage motif of the precursor HA is an exposed surface loop that presents the cleavage site to host proteases [67]. One potential limitation of our approach is that the peptides mimicking the cleavage sites may not form a loop structure and/or lack possible interactions between the precursor HA and host proteases required for proper cleavage, and may therefore produce results that are not observed with full length proteins. However, comparing our data with previous studies that examined the cleavage of precursor HA does not support this concern. Several studies have demonstrated that matriptase efficiently cleaves H1N1 and H9N2 HA and expresses low activity towards H2N2 and H3N2 HA subtypes [30,33,68]. KLK5 and KLK12 were shown to both cleave H1N1, H2N2 and H3N2 [31]. KLK12, however, had a higher affinity towards H1N1 and H2N2 compared to KLK5 while the latter cleaved H3N2 with a higher efficiency. It was also reported that plasmin cleaves H1N1 and WSN, expressing a higher affinity towards WSN than H1N1 [32]. Our results are therefore in line with these findings, with the single exception that we did not observe a significant difference in matriptase-mediated cleavage between H1N1 and H3N2 as described by Beaulieu et al. [33]. In addition, other studies found that HAT activates H1N1, H2N9 (which possess a IESR motif like H2N2), H3N2 and H9N2 virus [28,34,68] and that furin cleaves H5 HPAI [35]. None of these studies quantified the cleavage efficiency, but they support our interpretation that the cleavage we observe in our peptide assays reflects the situation with full-length HA. There is a discrepancy with one study that did not detect HAT mediated cleavage of H1 and H3 subtypes [27]. Our screen is, however, limited to soluble proteases and soluble active domains of TTSPs. TMPRSS2 and TMPRSS4 have been studied extensively and significantly contribute to the activation of IAV *in vivo* [28,29,34,69,70]. However, unless their purified active domains are available and shown to be soluble these and other TTSPs cannot be utilized in our peptide screen. There are limitations using the soluble catalytic domains of TTSPs which are discussed below.

To date, there have only been three IAV strains that have caused major pandemics—H1N1, H2N2 and H3N2 [3,42]. Our data suggests that the HA subtypes of all three strains are efficiently cleaved by a broad range of human proteases and that this broad spectrum contributed to the high pathogenicity of the H1N1, H2N2 and H3N2 strains. Besides H1N1, H2N2 and H3N2 we found a broad range cleavage only for H5N1 LPAI, H6N1 carrying the IETR motif and to some extent for H9N2. Both, the H5N1 LPAI and H6N1 IETR strains, currently have only limited potential for human infection, while H9N2 clearly has a higher potential [13,63,64]. Therefore, the adaption of the HA cleavage site towards human respiratory tract proteases alone is not sufficient to result in a pandemic IAV strain. In case of H1N1, H2N2 and H3N2 a number of biochemical and structural adaptations were necessary that allowed these strains to cross species barriers, to transmit between humans and to enter and to replicate efficiently in host cells [19,20]. Interestingly, HA of the A/Taiwan/2/2013 H6N1 strain, which was shown to have infected humans in the past [8], is cleaved less efficiently by the tested human proteases than HA from older avian isolates (Fig 3). Since the A/Taiwan/2/2013 H6N1 strain adapted to human sialic acid receptors and has proven to be able to infect humans we can only speculate that the less efficient cleavage of HA is a result of a natural variation in the cleavage site. In contrast, more relevant strains like H7N9 and H5N1 HPAI seem to be specifically activated by a single protease and are cleaved only very poorly or not at all by the other proteases tested in this panel. This also includes H10N8. In our assay only matriptase cleaved the H10 HA subtype very weakly but others demonstrated that TMPRSS2 cleaved up to 80% of the proteins in HA-expressing cells [27]. H7N9 and H9N2 were also reported to be cleaved by TMPRRS2 but cleavage was not quantified [68,69]. However, the study by Sakai et al., suggests that TMPRSS2 is the main protease activating H7N9 *in vivo* [69]. In conclusion it seems that a broad range of activating proteases assists the adaptation of an IAV strain to humans.

For our screen we used the catalytic domain of matriptase which expressed activity towards all tested HA subtype peptides except WSN. These results, however, come with a caveat and have to be interpreted carefully because they reveal a limitation of our assay using the catalytic domains of TTSPs. Matriptase is believed to localize to the basolateral side of epithelial cells where its catalytic activity is tightly regulated by the hepatocyte growth factor activator inhibitor type 1 (HAI-1) [71]. Cleavage of HAI-1 by a metalloprotease restores matriptase activity and leads to a shedding of the catalytic domain into the extracellular space [72]. Hamilton et al. showed that the catalytic domain was able to cleave the full length proteins of the H1, H2 and H3 subtypes expressed in mammalian cells as well as the peptide mimics used in this study [30]. It failed, however, to produce fusogenic versions of full length HA proteins of the H2 and H3 subtypes in cell fusion assays. Similar results were observed in a different study where the authors reported cleavage of H1 and H3 peptides by the catalytic matriptase domain but it only assisted the propagation of H1N1 virus in MDCK cells [33]. Therefore, the results achieved by our rapid screening method with active domains of TTSPs serve as an indicator but need to be validated by conventional methods. Other reports, however, support our data and validate our results. In addition to H1 isolates, matriptase was shown to cleave H9N2 virus and the report suggest that matriptase is the major activator of this strain *in vivo* [30,33,68].

One of the most interesting findings of our study was the efficient cleavage activation of H5N1 HPAI by matriptase. To date, it is believed that mainly furin is responsible for the cleavage of polybasic sites in HPAI strains which exacerbates the disease by causing a systemic infection [35,73]. The fact that matriptase expresses its highest affinity towards the H5N1 HPAI subtype peptide suggests a crucial role of this protease in the virulence of HPAI strains. However, matriptase is able to process Arg residues at the cleavage site at different positions and there is a possibility that the peptide is cleaved at the P1, P4 or P5 position [74]. Further experiments using full-length H5N1 HPAI HA are required to evaluate the contribution of matriptase to the fusogenic properties of this HA subtype.

The most effective measure against influenza is vaccination. Influenza vaccines are mostly produced in embryonated chicken eggs even though there are efforts to establish cell culture-based vaccine production [75,76]. There are certain limitations using eggs for vaccine production such as egg supply and low yield for some IAV strains which can become a significant issue for pandemic vaccine production [65,66,75]. Recent examples of poor viral growth in embryonated chicken eggs include the pandemic H1N1 virus from 2009 and the H3N2 A/Fujian/411/2002 strain which was selected for seasonal vaccine production in 2003/2004 [65,66]. Our data suggest that inefficient cleavage by Factor Xa significantly contributed to the low yield of these viruses. Mutation of the H1 cleavage site to resemble the preferred Factor Xa cleavage motif enhanced proteolytic processing about three-fold. There are examples where mutated versions of HA subtypes improved viral growth in chicken eggs without affecting antigenicity and immunogenicity [77–79]. None of these changes, however, was found in the cleavage site. It would be interesting to see if a modulation of the cleavage motif towards a sequence that is more effectively cleaved by Factor Xa improves viral growth in chicken eggs and at same time maintains the required antigenicity and immunogenicity. In contrast, H5 LPAI was shown to grow well in embryonated chicken eggs [80] but seems to be cleaved only very poorly by Factor Xa (Fig 7). One explanation could be that H5 HPAI is also cleaved very effectively by furin which is ubiquitously expressed in chicken embryos [81]. Therefore, H5 HPAI may not require Factor Xa for its cleavage and still grows well in chicken eggs.

In conclusion, our assay provides insights into the HA subtype specificity of different proteases located in human respiratory tract. Our data allow a rapid evaluation of the potential of a circulating strain to adapt to humans based on the cleavability of HA subtypes and can serve in combination with data regarding receptor specificity and interspecies transmissibility as powerful tool for pandemic risk assessment.

## Supporting information

S1 Table Vmax values of all proteases and corresponding HA substrates. (XLSX)
